# Systematic review of physical activity measurement scales validated for Arabic-speaking populations: insights from the BRIDGE project

**DOI:** 10.3389/fpubh.2026.1814479

**Published:** 2026-04-10

**Authors:** Hajar Mahfoudi, Soumaya Benmaamar, Meryeme Kirat, Ibtissam el Harch, Nassiba Bahra, Fatima Zahrae Bartal, Moncef Maiouak, Mohamed Aly, Osama Abdelkarim, Karima El Rhazi

**Affiliations:** 1 Department of Epidemiology, Clinical Research and Community Health, Faculty of Medicine, Pharmacy and Dentistry, Fez, Morocco; 2 Laboratory of Epidemiology and Research in Health Sciences, Sidi Mohamed Ben Abdellah University, Fez, Morocco; 3 Department of Epidemiology, Hassan II University Hospital Center, Fez, Morocco; 4 Faculty of Liberal Arts and Sciences, Chukyo University, Nagoya, Japan; 5 Faculty of Sport Sciences, Assiut University, Assiut, Egypt; 6 Department of Sports Management, School of Business, ESLSCA University, Giza, Egypt; 7 Institute of Sports and Sports Science, Karlsruhe Institute of Technology, Karlsruhe, Germany

**Keywords:** Arabic, COSMIN checklist, physical activity, psychometric properties, validation

## Abstract

**Background:**

Accurate assessment of physical activity (PA) is essential for epidemiological research and public health surveillance. In Arabic-speaking countries, most PA questionnaires originate from Western contexts, raising concerns about their cultural relevance and psychometric robustness when applied without proper validation.

**Objective:**

This systematic review aimed to identify PA measurement instruments available in Arabic and to critically appraise their psychometric properties using the COSMIN methodology.

**Methods:**

A systematic search was conducted in PubMed, Web of Science, and Scopus from inception to 2025, following PRISMA guidelines. Validation studies of PA questionnaires administered to Arabic-speaking populations were included. Data extraction and methodological quality assessment were independently performed by two reviewers. Psychometric properties were evaluated according to COSMIN criteria and classified as sufficient, insufficient, indeterminate, or variable.

**Results:**

A total of 21 studies were included, encompassing 13 different instruments across diverse populations and settings. The International Physical Activity Questionnaire (IPAQ) was the most frequently validated tool. Reliability and criterion validity were the most frequently assessed properties and generally demonstrated good methodological quality. However, criterion validity was often insufficient, particularly for widely used instruments such as the IPAQ and GPAQ. Structural validity, measurement error, responsiveness, and cross-cultural validity were rarely evaluated. Instruments designed for specific populations, such as older adults or individuals with disabilities, tended to show more favorable psychometric performance.

**Conclusion:**

Although several PA measurement instruments are available in Arabic, their psychometric evaluation remains heterogeneous and frequently incomplete. No instrument currently meets all COSMIN recommendations for robust psychometric quality. Future validation studies should adopt more comprehensive and methodologically rigorous approaches to improve the quality and comparability of PA measurement tools in Arabic-speaking contexts.

**Systematic review registration:**

https://www.crd.york.ac.uk/PROSPERO/view/CRD420261286986.

## Introduction

According to the WHO, PA includes any movement produced by skeletal muscles that requires energy consumption. This can include leisure activities, commuting, household chores, and work-related activities (1). Although often confused, the terms PA, exercise, and sport refer to distinct concepts (2, 3). A person may have a high level of PA due to their work, daily tasks, or active commuting, even without participating in sports or structured exercise programs (4).

PA is a major determinant of public health (5). It contributes to the prevention of certain non-communicable diseases (NCDs), particularly diabetes, hypertension, certain cardiovascular diseases, and many other chronic diseases (6). It also contributes to mental well-being, quality of life, and the reduction of premature mortality (1, 7, 8). Conversely, one of the main risk factors for premature mortality linked to NCDs is PA. The risk of premature mortality increases by 20 to 30% in inactive people compared to active people (1).

Increasing PA and fitness levels is associated with a reduction in the relative risk of death (by approximately 20 to 35%) (9, 10). A cohort of middle-aged men and women followed for 8 years revealed that individuals in the lowest quintiles of physical capacity, as assessed by treadmill testing, had a higher risk of death compared to those in the highest quintile (11).

Despite the well-established benefits of PA, nearly one-third of adults and the vast majority of adolescents (80%) do not meet recommended activity levels. International targets aim to reduce physical inactivity by 10% by 2025 and 15% by 2030. If no improvement is made, the cost to health systems could reach approximately $300 billion over the period 2020–2030 (1).

PA can be described in terms of several key dimensions. Frequency indicates how often the activity is performed. Intensity reflects the effort expended and the associated energy expenditure. Type of activity specifies the nature of the exercise and the expected physiological benefits. Finally, duration corresponds to the time spent on PA. Together, these parameters make it possible to accurately characterize active behavior (12).

Various methods are available to assess PA, encompassing both self-reported instruments such as the International Physical Activity Questionnaire (IPAQ), Global Physical Activity Questionnaire (GPAQ)…. (13), and objective measurement techniques including accelerometers and pedometers. Due to the high cost associated with objective methods of assessing PA, institutions most often turn to questionnaires, which are more accessible and easier to implement.

Most questionnaires originate from Western contexts. Their use in Arab countries can be problematic because cultural, linguistic, and social differences influence how questions are understood and how PA is measured. Therefore, simply translating a questionnaire is not enough: it must be carefully adapted to the local context and psychometrically evaluated to ensure that it actually measures what it is supposed to measure (14). For these reasons, several validation studies of PA measurement instruments have been conducted in the Arab context. The results of these studies vary considerably depending on the populations studied, the instrument validated, and the methods used. Even for popular questionnaires such as the IPAQ and the G-PAQ, validations sometimes yield inconsistent results, while other instruments intended for specific groups remain understudied (15–18).

To date, there has been no comprehensive overview of the PA measurement tools available in Arabic, nor any structured assessment of their psychometric properties. Given this situation, it is essential to carry out a systematic update in order to compile a list of validated instruments in Arabic and examine the rigor of their psychometric properties. Hence the objective of our study, which is to conduct a systematic review aimed at identifying all PA measurement instruments available in Arabic and critically evaluating their psychometric properties.

## Methods

### Study design

This systematic review was conducted in accordance with the PRISMA (Preferred Reporting Items for Systematic Reviews and Meta-Analyses) guidelines (19) and the COSMIN methodology for systematic reviews of patient-reported outcome measures (20).

### Search strategy

A systematic search was conducted in August 2025 in three databases: PubMed, Web of Science, and Scopus. The search covered all publications from inception to 2025, using following keywords and combined MeSH terms related to physical activity, validation and Arabic countries. For Web of Science and Scopus, the Boolean structure was as follows:

(“physical activity questionnaire” OR “physical activity scale” OR “physical activity tool”)

AND (“validation” OR “validity” OR “reliability” OR “translation” OR “cross-cultural adaptation” OR “psychometric properties” OR “reproducibility”).

For PubMed, a structured search using Medical Subject Headings (MeSH) and publication types was conducted. The strategy combined: “Exercise” [MeSH] AND “Surveys and Questionnaires” [MeSH] AND (“Validation Studies as Topic” [MeSH] OR “Validation Study” [Publication Type]) AND Geographic MeSH terms corresponding to Middle Eastern and North African countries and Arab populations. The detailed search strategies and equations for each database are provided in the Appendix 1.

In addition to the database searches, we manually examined the reference lists of all included studies to identify any further validation studies that might have been overlooked. This supplementary step did not yield any additional studies meeting the inclusion criteria.

Gray literature sources (e.g., theses, conference proceedings, institutional reports) were not systematically searched. The review focused exclusively on peer-reviewed full-text articles to ensure adequate methodological quality and comprehensive psychometric reporting.

### Eligibility criteria

Inclusion criteria.

Studies were included if they met the following criteria:

- Population: Arabic-speaking participants from Arab countries or Arabic-speaking communities abroad.
- Instrument: scales that measure PA, which have been translated or validated in Arabic.
- Type of study: validation studies, including or not translation/cultural adaptation procedures.
- Language of publication: articles published in English, French, or Arabic.
- Type of publication: full-text articles, peer-reviewed.

Exclusion criteria.

We excluded:

- Studies conducted outside Arab countries.
- Validations of instruments that did not measure PA.
- Studies addressing other concepts (e.g., nutrition, sedentary behavior not related to physical activity).
- Conference abstracts, theses, systematic reviews, study protocols, and other forms of gray literature.

### Studies selection

**Figure 1:**
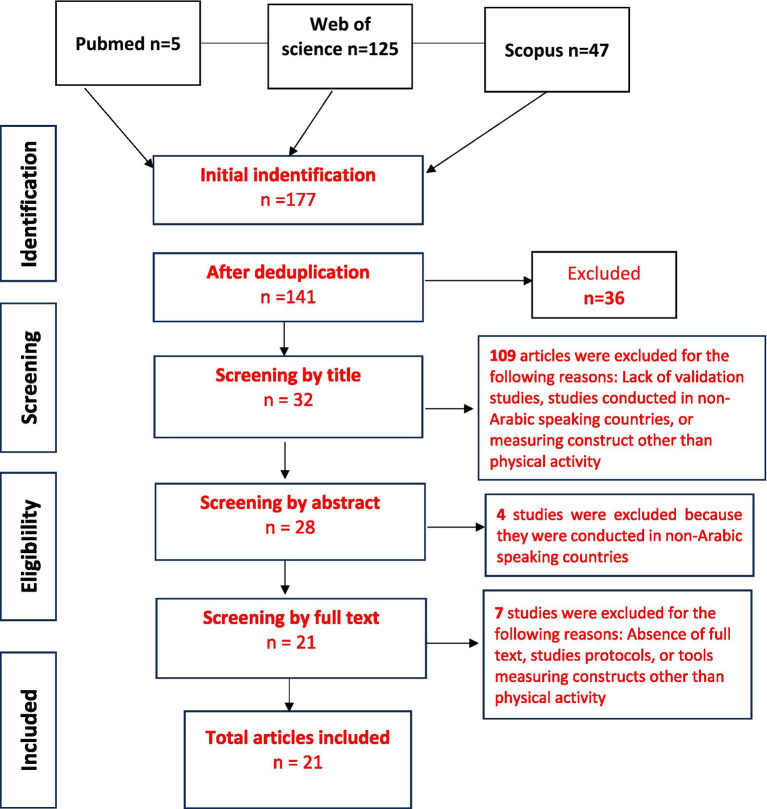
PRISMA flow diagram of studies selection process.

All identified articles were imported into Excel version 2013 so that duplicates could be removed. After removing duplicates, two reviewers independently examined the articles by title and then by abstract to determine whether they met the inclusion criteria. The two reviewers then read the selected articles in full. Disagreements were resolved through discussion or consultation with a third reviewer until consensus was reached. The studies selection process was documented in a PRISMA flow diagram Figure 1.

### Data extraction

A standardized extraction form was developed. For each study included, the following data were systematically extracted: the name of the instrument, the number of items and subscales, the country in which the validation was conducted, the original language of the tool, the target population, the sample size and the demographic characteristics of the participants (mean age, percentage of men), Information on the acceptability of the instrument and floor/ceiling effects, the translation and cultural adaptation process and the psychometric properties assessed (internal consistency, test–retest reliability, structural validity, construct validity, criterion validity, responsiveness, etc.). The extraction was performed independently by two evaluators.

In accordance with the COSMIN criteria for good measurement properties (21), each property was classified as:

- Sufficient (+) when the results met the COSMIN criteria.
- Insufficient (−) when they did not meet the established standards.
- Undetermined (?) when the information was missing, incomplete, or the methodology used did not allow for a conclusion.

When evaluating a multidimensional scale, each psychometric property was assessed separately for each dimension of the instrument. If all results for a given property across dimensions were sufficient, the property was rated as “sufficient.” If all results were insufficient, it was rated as “insufficient.” When results varied across dimensions, the property was rated as “Variable (+/−).”

This approach was used to simplify the presentation of results while still capturing differences in the measurement quality of each dimension. A summary of grading criteria and decision rules applied in this review is presented in Table 1.

**Table 1:** COSMIN measurement properties: definitions and rating criteria (20, 21)

Measurement property	Definition (20)	Rating	Criteria (20)
Content validity	The degree to which the content of a PROM is an adequate reflection of the construct to be measured	**+**	Included items are relevant for the construct, target population, and context of use, and response options and recall period are appropriate ***AND*** No key concepts are missing ***AND*** PROM items and response options are appropriately worded and PROM instructions, items and response options understood by the population of interest as intended
		**?**	Not enough information reported
		**−**	Included items are not relevant for the construct or target population ***OR*** Key concepts are missing ***OR*** PROM items and response options are not appropriately worded or not understood by the population of interest as intended
Structural validity	The extent to which the scores accurately reflect the expected factorial structure of the construct.	**+**	**CTT:** EFA/PCA: factor loadings of each item on its factor ≥0.30 ***AND*** Maximum 10% of the items have factor loadings ≥0.30 on multiple factors ***AND*** Explained variance *≥*50% and structure is in line with the theory about the construct to be measured *OR* results on scree plot or Kaiser criterion (Eigenvalues >1) are in line with the theory about the construct to be measured CFA: CFI or TLI or comparable measure >0.95 *OR* RMSEA <0.06 *OR* SRMR <0.08 **IRT/Rasch:** No violation of unidimensionality: CFI or TLI or comparable measure >0.95 *OR* RMSEA <0.06 *OR* SRMR <0.08 ***AND*** No violation of local independence: residual correlations among the items after controlling for dominant factor <0.20 *OR* Q3s < 0.37 ***AND*** No violation of monotonicity: adequate looking graphs *OR* item scalability >0.30 ***AND*** Adequate model fit: IRT: χ^2^ > 0.01 Rasch: infit and outfit mean squares ≥0.5 and ≤1.5 *OR* Z-standardized values > − 2 and <2
		**?**	Not enough information reported
		**−**	Criteria for ‘+’ not met
Internal consistency	The degree of the interrelatedness among the items	**+**	At least low evidence for sufficient unidimensionality AND Cronbach’s alpha ≥0.70
		**?**	Criteria for “at least low evidence for sufficient unidimensionality” not met ***OR*** Evidence for insufficient unidimensionality ***OR*** not enough information reported
		**−**	At least low-quality evidence for sufficient unidimensionality ***AND*** Cronbach’s alpha <0.70
Cross-cultural validity\ measurement invariance	The degree to which a translated/culturally adapted version accurately reflects the performance of the original version.	**+**	No important differences found between group factors (such as age, gender, language) in multiple group factor analysis ***OR*** no important DIF for group factors (McFadden’s R^2^ < 0.02)
		**?**	Not enough information reported
		**−**	Important differences between group factors ***OR*** DIF was found
Reliability	Stability of scores for a patient whose condition has not changed, assessed via test–retest, inter-rater, or intra-rater.	**+**	ICC or (weighted) kappa or Pearson/Spearman correlation ≥0.70
		**?**	Not enough information reported
		**−**	ICC or (weighted) kappa or Pearson/Spearman correlation <0.70
Measurement error	Systematic or random errors in scores that are not attributable to real changes in the construct.	**+**	SDC or LoA < MIC
		**?**	MIC not defined ***OR*** not enough information reported
		**−**	SDC or LoA > MIC
Criterion validity	Degree of correspondence between the instrument and an external reference considered to be the “gold standard.”	**+**	Correlation with gold standard ≥0.70 *OR* AUC ≥ 0.70
		**?**	Not enough information reported
		**−**	Correlation with gold standard <0.70 *OR* AUC < 0.70
Hypotheses testing for construct validity	The degree to which the scores are consistent with hypotheses, based on the assumption that the PROM validly measures the construct to be measured	**+**	≥75% of the results is in accordance with predefined hypotheses
		**?**	No relevant results were found
		**−**	≥75% of the results deviates from predefined hypotheses
Responsiveness	The ability of the instrument to detect changes over time in the measured construct.	**+**	≥75% of the results is in accordance with predefined hypotheses *OR* AUC ≥ 0.70
		**?**	No relevant results were found
		**−**	≥75% of the results deviates from predefined hypotheses *OR* AUC < 0.70

For multidimensional instruments, when the results differed across dimensions, the overall rating for that measurement property was classified as *Variable (+/−)*. AUC, area under the curve; CFA, confirmatory factor analysis; CFI, comparative fit index; CTT, classical test theory; DIF, differential item functioning; EFA, exploratory factor analysis; ICC, intraclass correlation coefficient; IRT, item response theory; LoA, limits of agreement; MIC, minimal important change; PCA, principal component analyses; RMSEA, Root Mean Square Error of Approximation; SEM, Standard Error of Measurement; SDC, smallest detectable change; SRMR, Standardized Root Mean Residuals; TLI, Tucker-Lewis index. Adapted with permission from (20), licensed under CC BY, https://doi.org/10.1007/s11136-018-1798-3.

### Quality assessment

The methodological quality of each of the included studies was assessed in accordance with the COSMIN (Consensus-based Standards for the selection of health Measurement Instruments) risk of bias checklist recommendations (22). For each study, the various methodological domains of the COSMIN Risk of Bias Checklist were examined, including: content validity, structural validity; internal consistency, cross-cultural validity\measurement invariance, reliability, measurement error, Criterion validity, hypotheses testing for construct validity and responsiveness.

Each domain was evaluated using the scoring system recommended by COSMIN, classifying the quality of the methodology as: “Very Good,” “Adequate,” “Doubtful,” “Inadequate,” or “Not applicable” With the lowest score among the elements in each domain being retained as the final score.

Sample size adequacy was assessed according to COSMIN standards as part of the methodological quality assessment. It was considered in the assessment of the quality of each study and was incorporated into the overall rating of the evidence.

### Ethical considerations

This review focuses on published articles; therefore, ethical approval was not required.

## Results

### Studies selection

A total of 141 articles were identified after deduplication from PubMed, Web of Science, and Scopus. Then after title screening, 32 articles were retained, while 109 were excluded mainly because they were not validation studies, they were conducted in non-Arabic-speaking countries, or assessed constructs other than PA. Following abstract screening, 28 articles remained, and 4 were excluded for similar reasons. Finally, after full-text screening, 21 studies were included in the review. The excluded papers at this stage mainly, were studies protocols, or assessed constructs unrelated to PA. One of these studies reported the validation of two different scales, which were both included in our analysis.

Figure 1 represent the studies selection process for our systematic review.

### Studies characteristics by measurement scale

Table 2 summaries the characteristics of the included studies by measurement scale.

**Table 2:** Characteristics of the included studies populations by country.

PROM	Country	Year of publication	Construct measured	Target population	Sample size (n)	Mean age (± SD)
IPAQ-SF	Jordan (23)	2011	Walking, MPA, VPA, sitting time/week and total PA	Healthy college students	194	20.4 ± 1.3
IPAQ-SF	UAE (25)	2024	walking, MPA, VPA, sitting time/week and total PA	Healthy young collegians adults	101	20.8 ± 2.4
IPAQ-SF	Saudi Arabia (26)	2025	MVPA	patients with pre-arthritic hip conditions	27	26.3 ± 9.3
IPAQ-SF	Jordan (24)	2021	Sedentary, MPA, VPA, MVPA	people with multiple sclerosis	50	36.7 ± 10.04
IPAQ-LF	Morocco (27)	2024	MPA, VPA, Walking, MVPA, Sitting, Total PA.	Healthy general population	120	33 ± 10
IPAQ-A	Tunisia (28)	2016	VPA, min/day MPA, min/day walking, min/day Total PA (MET), min/week	overweight or obese adolescents	51	16.8 ± 0.69
IPAQ-LF	Lebanon (15)	2017	walking, MPA, VPA, leisure PA, transportation PA, housework and house maintenance PA, occupational PA and total sitting time.	Lebanese general population	159	33.1 ± 12.9
IPAQ-SF	Kuwait (18)	2024	MVPA including walking MVPA excluding walking	diabetics type 1 and 2	n1 = 240 n2 = 343	44.77 ± 14.43/60.17 ± 10.77
PPAQ	Lebanon (29)	2020	Sedentary/ Moderate PA/ Vigorous PA	Pregnant women	179	29.94 ± 5.03
	Saudi Arabia (30)	2021	Vigorous intensity activity Moderate intensity activity Light intensity activity Sedentary activity total activity	Pregnant women	118	30.15 ± 5.59
PAQ-C	Morocco (31)	2023	MVPA	Children and adolescents	171	10.92 ± 1.55
	Saudi Arabia (32)	2022	MVPA	School-aged children	327	10.7 ± 1.3
ATLS	Saudi Arabia (33)	2011	Total time spent on all activities moderate-intensity activities vigorous-intensity activities specific activity: walking /jogging/swimming/house-hold/bicycling/martial arts/weight training	Adolescents	75	16.3 ± 1.2
	UAE (34)	2023	Sitting/sedentary behavior Walking Cycling High intensity activity Moderate intensity activity Total activity	Adolescents and young adults	131	20.47 ± 2.16
GPAQ	Saudi Arabia (35)	2016	Active commuting Vigorous exercise Moderate exercise Sedentary time	Young men	96	20 ± 1.1
	UAE (16)	2019	Sedentary, MPA, VPA, MVPA	Arabic speaking university students	93	21.27 ± 2.32
IPEQ	Jordan (24)	2021	MVPA	People with multiple sclerosis	50	36.7 ± 10.04
PASIPD-AR	Saudi Arabia (36)	2024	Not active at all Moderately active Active/extremely active	People with physical disabilities	206	39.66 ± 12.22
PASE	Saudi Arabia (37)	2018	Leisure time activity Household activity Work-related activity Total PASE score	Older adults	74	65.0 ± 7.1
GSLTPAQ	Saudi Arabia (38)	2022	strenuous, moderate, and minimal exercise	Office Workers	150	46.64 ± 10.24
PAQ-A	Saudi Arabia (17)	2023	Moderately PA vigorously PA.	Adolescents girls	383	15.43 ± 1.68
QAPACE	UAE (39)	2012	Sleeping time weekend day Walking time at school Running time at school Total active time during weekday	Children	79	-

MVPA, moderate to vigorous physical activity; VPA, Vigorous physical activity; MPA, Moderate physical activity; Total PA, total physical activity; PA, physical activity; IPAQ-SF, International physical activity questionnaire-Short Form; IPAQ-LF, International physical activity questionnaire-Long Form; IPAQ-adolescents, International physical activity questionnaire for adolescents; PPAQ, pregnancy Physical Activity Questionnaire; PAQ-C, physical activity questionnaire for children; ATLS, Arab Teens Lifestyle Study physical activity; GPAQ, Global Physical Activity Questionnaire; IPEQ, Incidental and Planned Exercise Questionnaire; PASIPD-AR, Physical Activity Scale for Individuals with Physical Disabilities; PASE, Physical Activity Scale for the Elderly; GSLTPAQ, Godin Shephard Leisure-Time Physical Activity Questionnaire; PAQ-A, Physical Activity Questionnaire for Adolescents; QAPACE, Quantification de l’Activité Physique en Altitude Chez les Enfants; UAE, United Arab Emirates.

#### International physical activity questionnaire

The IPAQ was the most frequently used instrument among the included studies. It was applied across several Arabic countries, including Jordan (23, 24), the UAE (United Arab Emirates) (25), Saudi Arabia (26), Morocco (27), Tunisia (28), Lebanon (15) and Kuwait (18). The tool was administered to highly heterogeneous population, ranging from healthy individuals to specific clinical groups such as people with multiple sclerosis, patients with type 1 and type 2 diabetes, and individuals with pre-arthritic hip conditions. Sample sizes varied widely, from fewer than 27 participants to more than 300, and mean ages spanned from 16.8 ± 0.69 to 60.17 ± 10.77.

#### The pregnancy physical activity questionnaire

The PPAQ was used exclusively among pregnant women in studies conducted in Lebanon (29) and Saudi Arabia (30). These studies involved moderate sample sizes ranging from 118 to 179, with mean participant ages around 30 years (29.94 ± 5.03 for Lebanon and 30.15 ± 5.59 for Saudi Arabia).

#### The physical activity questionnaire for children

The PAQ-C was administered in Morocco (31) and Saudi Arabia (32) among school-aged children and adolescents. The included studies reported relatively large sample sizes (171 in the Moroccan study and 327 in the Saudi Arabian study), with mean ages clustered around 10 years (10.92 ± 1.55 and 10.7 ± 1.3, respectively).

#### The Arab teens lifestyle study physical activity

ATLS has been used in Saudi Arabia (33) and the UAE (United Arab Emirates) (34) among adolescents and young adults. Sample sizes are intermediate (75 and 131 respectively), and average ages ranging from late adolescence (16.3 ± 1.2) to early adulthood (20.47 ± 2.16).

#### The global physical activity questionnaire

The GPAQ has been administered to populations of young adults, particularly young men and Arabic-speaking university students, in Saudi Arabia (35) and the UAE (United Arab Emirates) (16). Sample sizes were moderate (96 for Saudi Arabia and 93 for UAE), and the average ages was around 20 years (20 ± 1.1 and 21.27 ± 2.32 respectively).

#### The incidental and planned exercise questionnaire

The IPEQ has been validated only in people with multiple sclerosis in Jordan (24). The corresponding study has a limited sample size (*n* = 50) and an average age of 36.7 ± 10.04.

#### The physical activity scale for individuals with physical disabilities

The PASIPD-AR has been validated in Saudi Arabia (36), in people with physical disabilities. The sample size was relatively large (*n* = 206) and the average age was 39.66 ± 12.22.

#### The physical activity scale for the elderly

The PASE has been validated exclusively in older adults in Saudi Arabia (37). Participants had a high average age (over 65.0 ± 7.1), and a sample size of 74 participants.

#### The Godin Shephard leisure-time physical activity questionnaire

The GSLTPAQ was validated in Saudi Arabia (38) with office workers. The corresponding study includes a moderate-sized sample (150) composed exclusively of middle-aged adults, with an average age of around 46.64 ± 10.24.

#### The physical activity questionnaire for adolescents

The PAQ-A was validated in Saudi Arabia (17) to a large sample (383) of adolescent girls. The average age of participants was 15.43 ± 1.68.

#### The quantification de l’activité physique en altitude chez les enfants

In the United Arab Emirates (39), the QAPACE was validated in a pediatric population comprising 79 children’s.

### Quality assessment by psychometric property

Table 3 summarizes the methodological quality of the included studies based on the COSMIN checklist. Overall, the results show that certain measurement properties have been frequently evaluated and are mostly good to very good methodological quality, while other areas remain largely unexplored.

**Table 3:** Methodological quality assessment.

Psychometric property	Number of studies	Very good N (%)	Adequate N (%)	Doubtful N (%)	Inadequate N (%)	Not applicable	Main statistical tests used
Internal consistency	7	7 (100%)	0	0	0	15	Cronbach’s alpha
Reliability	12	9 (75%)	1 (8.3%)	2 (16.7%)	0	10	ICC/spearman CC
Criterion validity	14	13 (92.9%)		0	1 (7.1%)	8	Pearson’s CC/ spearman’s CC
Structural validity	4	3 (75%)	1 (25%)	0	0	18	EFA/CFA
Cross-cultural validity/measurement invariance	0	0	0	0	0	22	-
Measurement error	2	2 (100%)	0	0	0	20	SEM/MDC
Hypotheses testing for construct validity	10	8 (80%)	0	0	2 (20%)	12	Pearson’s CC/ spearman’s CC
Responsiveness	0	0	0	0	0	22	-
Content validity	3	0	3 (100%)	0	0	19	-

ICC, Intra class Correlation Coefficient; Pearson’s CC, Pearson’s Correlation Coefficient; Spearman’s CC, Spearman’s Rank Correlation Coefficient; EFA, Exploratory Factor Analysis; CFA, Confirmatory Factor Analysis; SEM, Standard Error of Measurement; MDC, Minimal Detectable Change.

#### Internal consistency

Internal consistency was examined in 7 studies. All of them (100%) were rated as having very good methodological quality. The majority of analyses were based on Cronbach’s alpha coefficient, indicating good internal consistency of the instruments evaluated. This property was not applicable in 15 studies.

#### Reliability

Reliability was analyzed in 12 studies. Of these, 9 studies (75%) were rated as very good quality, one study (8.3%) was rated as adequate, and two studies (16.7%) were rated as doubtful. No studies were rated as inadequate. The analyses were based mainly on the intraclass correlation coefficient (ICC) and Spearman’s correlation coefficient. This property was not applicable in 10 studies.

#### Criterion validity

Criterion validity is one of the most frequently and rigorously evaluated properties, with 14 studies concerned. 13 studies (92.9%) were judged as very good quality, while one study (7.1%) was judged to be inadequate. The statistical tests used included Pearson’s and Spearman’s correlation coefficients. This property was not applicable in 8 studies.

#### Structural validity

Structural validity was assessed in only four studies. Three studies (75%) were rated as very good quality, and one study (25%) was rated as adequate quality. The analyses were based on exploratory and confirmatory factor analyses (EFA/CFA). This property was not applicable in 18 studies.

#### Cross-cultural validity/measure invariance

No studies (0 studies) evaluated cross-cultural validity or measure invariance.

#### Measurement error

Measurement error was assessed in only 2 studies, both of which (100%) were considered to be as very good methodological quality, using SEM (Standard Error of Measurement) and MDC (Minimal Detectable Change). This property was not applicable in 20 studies.

#### Hypothesis testing for construct validity

Hypothesis testing for construct validity was performed in 10 studies. 8 studies (80%) were rated as very good quality, while 2 studies (20%) were rated as inadequate. The analyses were mainly based on Pearson and Spearman correlation coefficients. This property was not applicable in 12 studies.

#### Responsiveness

No studies assessed the responsiveness of the instruments.

#### Content validity

Content validity was assessed in three studies, all (100%) of which were deemed to be of adequate quality, with no studies classified as very good or inadequate.

### Psychometric results by measurement scale

The psychometric properties of the included PA measurements instruments are summarized in Table 4, the results vary between tools and between countries.

**Table 4:** Psychometric results by measurement scale.

PROM	Country	Internal consistency	Reliability	Criterion validity	Gold standard (reference test)	Structural validity	Hypotheses testing	Measurement error
IPAQ-SF	Jordan (23)	NA	+	NA	NA	NA	−	NA
IPAQ-SF	UAE (25)	NA	NA	−	Accelerometer	NA	NA	NA
IPAQ-SF	Saudi Arabia (26)	NA	NA	−	Accelerometer	NA	+/−	NA
IPAQ-SF	Jordan (24)	NA	NA	−	Actigraph	NA	+/−	NA
IPAQ-LF	Morocco (27)	NA	+/−	−	Accelerometer	NA	NA	NA
IPAQ-A	Tunisia (28)	NA	+	−	Pedometer	NA	NA	NA
IPAQ-LF	Lebanon (15)	+	+/−	NA	NA	−	+/−	NA
IPAQ-SF	Kuwait (18)	NA	NA	−	Accelerometer	NA	NA	NA
PPAQ	Lebanon (29)	NA	NA	NA	NA	NA	NA	NA
	Saudi Arabia (30)	+/−	+	NA	NA	NA	NA	NA
PAQ-C	Morocco (31)	NA	+/−	−	Accelerometer	NA	NA	NA
	Saudi Arabia (32)	+	NA	NA	NA	EFA− CFA+	NA	NA
ATLS	Saudi Arabia (33)	NA	NA	−	Pedometer	NA	+	NA
	UAE (34)	NA	NA	−	Accelerometer	NA	+	NA
GPAQ	Saudi Arabia (35)	NA	+/−	−	Accelerometer	NA	−	NA
	UAE (16)	NA	+/−	−	Accelerometer	NA	NA	NA
IPEQ	Jordan (24)	NA	NA	−	Actigraph	NA	−	NA
PASIPD-AR	Saudi Arabia (36)	+/−	+	NA	Na	EFA+ CFA+	NA	?
PASE	Saudi Arabia (37)	+	+	NA	NA	NA	+/−	?
GSLTPAQ	Saudi Arabia (38)	+	+	+	CCHSLTPAQ	NA	NA	NA
PAQ-A	Saudi Arabia (17)	+	NA	NA	NA	−	−	NA
QAPACE	UAE (39)	NA	−	+/−	pedometer	NA	NA	NA

Sufficient (+) when the results met the COSMIN criteria. Insufficient (−) when they did not meet the established standards. Undetermined (?) when the information was missing, incomplete, or the methodology used did not allow for a conclusion. +/−: variable Results between dimensions. IPAQ-SF, International physical activity questionnaire-Short Form; IPAQ-LF, International physical activity questionnaire-Long Form; IPAQ-adolescents, International physical activity questionnaire for adolescents; PPAQ, pregnancy Physical Activity Questionnaire; PAQ-C, physical activity questionnaire for children; ATLS, Arab Teens Lifestyle Study physical activity; GPAQ, Global Physical Activity Questionnaire; IPEQ, Incidental and Planned Exercise Questionnaire; PASIPD-AR, Physical Activity Scale for Individuals with Physical Disabilities; PASE, Physical Activity Scale for the Elderly; GSLTPAQ, Godin Shephard Leisure-Time Physical Activity Questionnaire; PAQ-A, Physical Activity Questionnaire for Adolescents; QAPACE, Quantification de l’Activité Physique en Altitude Chez les Enfants; UAE, United Arab Emirates. CCHSLTPAQ, Copenhagen City Heart Study Leisure Time Physical Activity Questionnaire.

#### The international physical activity questionnaire

The IPAQ was the most frequently validated instrument among Arabic-speaking populations. However, the psychometric results were not always consistent or strong. Internal consistency was largely not assessed, with the exception of the Lebanese study (15), in which sufficient internal consistency (+) was reported. Reliability showed variable quality across countries, sufficient reliability (+) was observed in studies conducted in Jordan (23) and Tunisia (28), whereas in Morocco (27) and Lebanon (15) the reliability was considered variable (+/−). Criterion validity consistently demonstrated insufficient results (−) in all studies where it was assessed. Structural validity was rarely examined and, when assessed in Lebanon (15), yielded insufficient results (−). Construct validity, assessed through hypothesis testing, showed mixed findings, variable results (+/−) were reported in studies from Jordan (24), Saudi Arabia (26), and Lebanon (15), and insufficient result (−) was reported in another study conducted in Jordan (23). Finally, measurement error was not evaluated in any of the included studies.

#### The pregnancy physical activity questionnaire

In the Lebanese study (29), psychometric properties were not evaluated; therefore, the psychometric quality of the PPAQ in this context cannot be determined. In contrast, the Saudi Arabian study (30) provided partial psychometric evidence. Internal consistency yielded variable results (+/−), whereas reliability was rated as sufficient (+).

#### The physical activity questionnaire for children

The Moroccan (31) validation presented limited results, with only variable reliability (+/−) and weak criterion validity (−). However, in Saudi Arabia (32), the PAQ-C showed sufficient internal consistency (+), and the structural validity was confirmed using Confirmatory Factor Analysis (+).

#### The Arab teens lifestyle study physical activity

Was evaluated in Saudi Arabia (33) and the UAE (34), showing limited evidence for reliability, insufficient criterion validity (−), and sufficient hypotheses testing (+).

#### The global physical activity questionnaire

Demonstrated mixed results (+/−) on reliability and insufficient criterion validity (−) in Saudi Arabia (35) and the UAE (16), with limited assessment of other properties. For hypothesis testing the Saudi Arabia (35) study showed insufficient result (−).

#### The incidental and planned exercise questionnaire

The study (24) reported insufficient criterion validity (−) and hypothesis testing (−), whereas the other psychometric properties were not evaluated.

#### The physical activity scale for individuals with physical disabilities

The PASIPD-AR in Saudi Arabia (36) showed variable internal consistency (+/.) and suffcient reliability (+), with good structural validity (EFA+, CFA+), whereas the measurement error was indeterminate (?).

#### The physical activity scale for the elderly

Showed sufficient internal consistency and reliability (+), with mixed results in hypotheses testing (+/−) and unclear measurement error (?).

#### The Godin Shephard leisure-time physical activity questionnaire

The study conducted in Saudi Arabia (38) demonstrated consistently sufficient internal consistency, reliability, and criterion validity (+).

#### The physical activity questionnaire for adolescents

The PAQ-A in Saudi Arabia (17) showed sufficient internal consistency (+), insufficient structural validity (−), and insufficient hypotheses testing (−).

#### The quantification de l’activité physique en altitude chez les enfants

The QAPACE in the UAE (39) demonstrated insufficient reliability (−) and mixed criterion validity (+/−).

### Psychometric performance according to target populations

The studies included in this review evaluated physical activity questionnaires across a wide range of populations, including children and adolescents, adults, older individuals, and people living with specific health conditions. In addition, a distinction was observed between generic questionnaires designed for use in the general population and instruments developed for particular demographic or clinical groups.

Generic questionnaires, such as the International Physical Activity Questionnaire (IPAQ) and the Global Physical Activity Questionnaire (GPAQ), were the most frequently investigated tools and were predominantly evaluated among adult populations. When reliability was examined, these instruments generally showed acceptable levels of consistency. However, their criterion validity was often reported as insufficient when compared with objective reference measures, including accelerometers or pedometers.

Several questionnaires were specifically designed for particular target populations. Instruments developed for younger individuals, such as the Physical Activity Questionnaire for Children (PAQ-C), the Physical Activity Questionnaire for Adolescents (PAQ-A), and the Arab Teens Lifestyle Study questionnaire (ATLS), provided some evidence supporting structural validity, although other measurement properties were less frequently examined. Likewise, questionnaires developed for specific conditions or life stages, including the Pregnancy Physical Activity Questionnaire (PPAQ), the Physical Activity Scale for the Elderly (PASE), and the Physical Activity Scale for Individuals with Physical Disabilities (PASIPD), showed heterogeneous psychometric results depending on the property assessed.

Overall, although generic questionnaires have been more widely studied, evidence regarding their criterion validity remains inconsistent. In contrast, population-specific instruments have been evaluated in fewer studies, leading to more limited but potentially more relevant psychometric evidence for the populations for which they were originally developed.

## Discussion

This systematic review aimed to identify all PA measurement instruments available in Arabic and to critically evaluate their psychometric properties using a uniform framework, namely the COSMIN methodology. A total of 21 studies were included, covering a wide range of instruments, target populations, and clinical or community settings. Most studies were conducted in Gulf countries, particularly in Saudi Arabia and the United Arab Emirates. The (IPAQ) was the most frequently validated instrument.

The assessment of methodological quality, based on the COSMIN checklist, showed that criterion validity, reliability, and construct validity were the most frequently examined psychometric properties. These properties generally demonstrated good to very good methodological quality; however, several key psychometric dimensions remain largely underexplored. Overall, the evaluation of psychometric properties varied considerably across studies, reflecting substantial heterogeneity in the reported results. Importantly, the adequacy of reported psychometric results did not always correspond to methodological ratings.

The apparent discrepancy between high methodological quality and insufficient psychometric results warrants clarification. Within the COSMIN framework, risk-of-bias assessments evaluate the rigor of study design and the statistical methods used to assess a measurement property. In contrast, the rating of results reflects adherence to predefined adequacy criteria (e.g., correlation thresholds). Therefore, a study may be methodologically rigorous while still demonstrating insufficient psychometric performance.

Among all the identified instruments, the (IPAQ) is the most frequently validated in Arab countries. It has been evaluated in several contexts including Jordan, Saudi Arabia, the United Arab Emirates, Morocco, Tunisia, Lebanon, and Kuwait, reflecting its widespread use and its status as a reference tool for PA measurement. These findings are consistent with international reviews, which indicate that the IPAQ is one of the most widely used and validated questionnaires worldwide (40). IPAQ was applied to different age groups including (young’s and adolescents, healthy general population of adults) and to different clinical settings including (patients with pre-arthritic hip conditions, people with multiple sclerosis and diabetics type 1 and 2). Although the most frequently used tool (40), has limited psychometric performance overall, particularly in terms of criterion validity, which proved insufficient in all the studies included. These results are consistent with international reviews, which also report weak to moderate correlations between the IPAQ and objective measures such as accelerometers (41, 42). This confirms that the popularity and widespread use of the IPAQ do not necessarily guarantee optimal psychometric quality.

Similarly, the GPAQ, although recommended, has shown insufficient criterion validity and variable reliability results in studies conducted in Saudi Arabia (35) and the United Arab Emirates (34). These observations are consistent with the conclusions of international reviews indicating that the criterion validity of self-reported PA questionnaires remains moderate overall, even in contexts where more rigorous methodologies are used (43).

However, certain instruments specific to target populations, such as the GSLTPAQ, PASE, and PASIPD-AR, have shown relatively more satisfactory psychometric results, particularly in terms of reliability and internal consistency. Which suggests that questionnaires designed for specific groups such as PASE in older adults, often perform better than generic tools, probably because they are better suited to the functional context of the respondents, as has been observed in international systematic reviews (44).

In children and adolescents, the results observed for the PAQ-C, PAQ-A, ATLS, and QAPACE reveal heterogeneous and often incomplete psychometric performance. These results are comparable to those reported in international journals devoted to young populations, which also highlight a lack of solid evidence regarding construct validity and test–retest reliability, of many instruments (45). This heterogeneity can be explained in part by the developmental characteristics of children and adolescents, particularly their limited ability to accurately recall and report the intensity and frequency of PA. In addition, methodological weaknesses, such as the lack of hypothesis formulation and suboptimal study designs, also contribute to the inconsistent psychometric evidence observed for these instruments.

The studies included in this review revealed several limitations related to the assessment of psychometric properties. Many validation studies reported incomplete psychometric evaluations. The limited and heterogeneous evidence regarding validity properties restricts the interpretability and comparability of results across studies. This methodological heterogeneity is consistent with the conclusions of international reviews devoted to physical activity questionnaires. Indeed, Silsbury et al. (43), Mireille et al (46) and other systematic reviews (44, 45) have shown that, even in Western contexts, most studies focus on a limited number of psychometric properties, often to the detriment of a comprehensive assessment as recommended by COSMIN.

Key properties such as construct validity, structural validity, and measurement error frequently not assessed, despite their importance for ensuring that an instrument accurately measures PA and can detect meaningful changes over time. Reliability-related properties, including internal consistency and test–retest reliability, were more frequently reported and generally showed acceptable results, highlighting the usefulness of these measures for assessing the stability of self-reported PA.

A particularly important finding of this review is that none of the included studies assessed cross-cultural validity or responsiveness. According to the COSMIN standards (20), cross-cultural validity is essential when an instrument is translated or used in different linguistic and cultural contexts, as it ensures that the construct is interpreted equivalently across groups. The absence of such evaluation raises concerns about the comparability of results across populations and limits confidence in pooled or cross-national analyses.

Similarly, responsiveness defined by the COSMIN framework (20) as the ability of an instrument to detect change over time was not examined in any study. This represents a substantial limitation, particularly if the instrument is intended for longitudinal research, intervention studies, or educational program evaluation. Without evidence of responsiveness, it remains uncertain whether observed score changes reflect true changes in the construct or merely measurement variability.

This gap highlights a priority for future research: well-designed studies assessing measurement invariance across cultures and longitudinal sensitivity to change, using robust statistical approaches such as multi-group confirmatory factor analysis and effect size–based responsiveness indices.

This systematic review has several strengths, including adherence to PRISMA and COSMIN guidelines, the use of multiple databases, independent screening and data extraction by two reviewers, and a structured evaluation of multiple psychometric properties. Nevertheless, the findings confirm that no PA measurement instrument currently available in Arabic can be considered fully robust across all recommended psychometric dimensions. This underscores the need for future validation studies adopting more comprehensive and methodologically rigorous approaches to improve the quality, robustness, and comparability of PA measurement tools in Arabic-speaking contexts.

## Conclusion

This systematic review shows that, although several PA measurement instruments are available in Arabic, their psychometric properties remain heterogeneous and often incompletely assessed. The most widely used questionnaires, such as the IPAQ and the GPAQ, present notable limitations, particularly in terms of criterion validity. Furthermore, none of the included studies assessed cross-cultural validity or responsiveness, despite the fact that many of these instruments were initially developed in Western contexts and then translated into Arabic. Instruments specifically developed for certain populations appear to demonstrate better performance, highlighting the importance of adapting measurement tools to the context of the respondents. Overall, no instrument currently available in Arabic can be considered fully robust according to COSMIN recommendations, underscoring the need for future validation studies adopting more rigorous and comprehensive methodological approaches in Arabic-speaking contexts.

## Data Availability

The original contributions presented in the study are included in the article/Supplementary material, further inquiries can be directed to the corresponding author.
