# Supplementary material for: Systematic review of physical activity measurement scales validated for Arabic-speaking populations: insights from the BRIDGE project
Source: Front Public Health. 2026 Apr 10;14:1814479. doi: 10.3389/fpubh.2026.1814479 (PMC13108486; doi:10.3389/fpubh.2026.1814479)
Supplement: Supplementary file 1 [file Supplementary_file_1.docx]

**Supplementary Table 1.** Search strategy used in Web of Science, Scopus, and PubMed to identify studies on physical activity questionnaires.

**Web of science and scopus**

(("physical activity questionnaire" OR " physical activity scale" OR "physical activity tool " ) AND ( "validation" OR "validity" OR "reliability" OR "translation" OR "cross-cultural adaptation" OR "psychometric properties" OR "reproducibility" ) )

**Pub med**

(("Exercise"[Mesh]) AND "Surveys and Questionnaires"[Mesh]) AND ( "Validation Studies as Topic"[Mesh] OR "Validation Study" [Publication Type] ) AND ("Middle East"[Mesh] OR "Africa, Northern"[Mesh] OR "Arabs"[Mesh] OR " Afghanistan "[Mesh] OR "Algeria"[Mesh] OR "Bahrain"[Mesh] OR "Djibouti"[Mesh] OR "Egypt"[Mesh] OR "Iran"[Mesh] OR "Iraq"[Mesh] OR "Jordan"[Mesh] OR "Kuwait"[Mesh] OR "Lebanon"[Mesh] OR "Libya"[Mesh] OR "Morocco"[Mesh] OR "Oman"[Mesh] OR "Pakistan"[Mesh] OR "Palestine"[Mesh] OR "Qatar"[Mesh] OR "Saudi Arabia"[Mesh] OR "Somalia"[Mesh] OR "Sudan"[Mesh] OR "Syria"[Mesh] OR "Tunisia"[Mesh] OR "United Arab Emirates"[Mesh] OR "Yemen"[Mesh])
